# Early *BCR-ABL1* Transcript Decline after 1 Month of Tyrosine Kinase Inhibitor Therapy as an Indicator for Treatment Response in Chronic Myeloid Leukemia

**DOI:** 10.1371/journal.pone.0171041

**Published:** 2017-01-30

**Authors:** Mohamed El Missiry, Henrik Hjorth-Hansen, Johan Richter, Ulla Olson-Strömberg, Leif Stenke, Kimmo Porkka, Anna Kreutzman, Satu Mustjoki

**Affiliations:** 1 Hematology Research Unit Helsinki, Department of Hematology, University of Helsinki and Helsinki University Central Hospital Comprehensive Cancer Center, Helsinki, Finland; 2 Department of Hematology, St Olavs Hospital, Trondheim, Norway; 3 Department of Cancer Research and Molecular Medicine, St Olavs Hospital, Norwegian University of Science and Technology (NTNU), Trondheim, Norway; 4 Department of Hematology and Vascular Disorders, Skåne University Hospital, Lund, Sweden; 5 Department of Hematology, Uppsala University Hospital, Uppsala, Sweden; 6 Department of Hematology, Karolinska University Hospital and Karolinska Institutet, Stockholm, Sweden; 7 Department of Clinical Chemistry, University of Helsinki, Helsinki, Finland; Universita degli Studi di Firenze, ITALY

## Abstract

In chronic myeloid leukemia (CML), early treatment prediction is important to identify patients with inferior overall outcomes. We examined the feasibility of using reductions in *BCR-ABL1* transcript levels after 1 month of tyrosine kinase inhibitor (TKI) treatment to predict therapy response. Fifty-two first-line TKI-treated CML patients were included (imatinib n = 26, dasatinib n = 21, nilotinib n = 5), and *BCR-ABL1* transcript levels were measured at diagnosis (dg) and 1, 3, 6, 12, 18, 24, and 36 months. The fold change of the *BCR-ABL1* transcripts at 1 month compared to initial *BCR-ABL1* transcript levels was used to indicate early therapy response. In our cohort, 21% of patients had no decrease in *BCR-ABL1* transcript levels after 1 month and were classified as *poor responders*. Surprisingly, these patients had lower *BCR-ABL1* transcript levels at dg compared to *responders* (31% vs. 48%, p = 0.0083). *Poor responders* also significantly more often had enlarged spleen (55% vs. 15%; p<0.01) and a higher percentage of Ph+ CD34+CD38- cells in the bone marrow (91% vs. 75%, p<0.05). The major molecular response rates were inferior in the *poor responders* (at 12m 18% vs. 64%, p<0.01; 18m 27% vs. 75%, p<0.01; 24m 55% vs. 87%, p<0.01). In conclusion, early treatment response analysis defines a biologically distinct patient subgroup with inferior long-term outcomes.

## Introduction

Chronic myeloid leukemia (CML) is caused by the Philadelphia chromosome (Ph), which induces the formation of the BCR-ABL1 fusion protein. It has constant tyrosine kinase activity leading to uncontrolled cell proliferation.[1–3] Tyrosine kinase inhibitors (TKIs) blocking the BCR-ABL1 oncokinase activity have revolutionized the treatment and prognosis of CML. The first-generation TKI imatinib has been followed by the second-generation inhibitors dasatinib and nilotinib, which induce faster and deeper molecular responses.[4–6] The current treatment goal of TKI therapy is to achieve first a major molecular response (MMR; *BCR-ABL1* transcripts<0.1% in the International Scale) and later deeper molecular responses, such as MR4.0 and MR4.5. According to the 2013 European Leukemia Net (ELN) recommendations, MMR is expected to be reached by 12 months,[7] as it has been shown that patients who have achieved this treatment goal have significantly better overall survival.[4,7–9] The 12-month MMR rates for nilotinib and dasatinib have been reported to be between 45% and 81%, whereas with imatinib the MMR rates are generally lower.[10,11]

Even though the majority of CML patients achieve very good therapy responses, a small fraction of patients does not respond to the assigned TKI. Therefore, early prediction of the treatment response remains one of the major focuses for researchers as well as clinicians, in order to find the ideal time to increase the dose or switch the type of TKI to achieve optimal long-term outcome and avoid relapse. Several methods to predict treatment responses have been established either by using diagnostic scores (Sokal, Euro, or EUTOS scores) or by assessing the level of leukemic cell burden via cytogenetics or molecular genetics during treatment.[7,12–14] These days, the *BCR-ABL1* mRNA molecular assessments are the most accurate and common way to evaluate response. Recently, it has been shown that the evaluation of early treatment response at 3 months may be of importance.[15–18] Several studies have indicated that patients with a *BCR-ABL1* transcript level lower than 10% at 3 months have an overall survival rate of 95% in comparison with 85% in patients above this level.[7,15,16,19]

As the treatment responses to TKIs are generally very fast, we hypothesized that the prediction of overall treatment response could be done earlier than 3 months, particularly with the emergence of newer TKI compounds. Therefore, in this study, we have analyzed the initial decline of *BCR-ABL1* transcript levels during the first month of therapy and its importance for later achievement of optimal treatment responses.

## Patients and Methods

### Study patients

A total of 52 newly diagnosed chronic phase CML patients from the Nordic countries (Finland, Sweden, and Norway) were included in this study. Of them, 26 patients started on imatinib, 21 on dasatinib, and 5 on nilotinib as first-line therapy. The patients had participated in either the NordCML006 (NCT00852566) or the ENESTnd (NCT00471497) clinical trials,[11,20] and the only selection criterion was that both diagnostic phase and 1-month PCR values should be available for analysis. Patients provided written informed consent before the start of the trial. The study was approved by the Helsinki University Central Hospital ethics committee with the consideration of the principles of the Helsinki Declaration.

### Molecular and cytogenetic response analysis

Molecular genetic analysis was performed with real-time quantitative PCR (RQ-PCR) analyses to detect the amount of *BCR-ABL1* transcripts in the peripheral blood cells by TaqMan® chemistry. RT reaction conditions and RQ-PCR assays were performed according to the protocol of the Europe Against Cancer (EAC) Program [21], using either *ABL* or *GUS* as reference genes. The same reference gene was used in all individual patients throughout the follow-up. The *BCR-ABL1* transcript values were reported in the international scale (*BCR-ABL*^IS^).[22] Molecular treatment responses were evaluated at diagnosis (dg) and 1, 3, 6, 12, 18, 24, and 36 months after the start of TKI treatment. Karyotyping of bone marrow (BM) cells was performed using standard G-banding analysis of at least 20 metaphases at dg and 1, 3, and 6 months after therapy started.

### Cell sorting and fluorescence in situ hybridization (FISH)

Mononuclear cells from BM samples were separated using Ficoll centrifugation (GE Healthcare Bio-Sciences AB, Uppsala, Sweden). CD34 positive (CD34+) cells were further enriched using paramagnetic beads (Miltenyi Biotech, Bergisch Gladbach, Germany), and they were stained after separation with fluorescence-conjugated CD34 and CD38 antibodies (BD Biosciences, San Jose, CA, USA). CD34+ cells were sorted with FACS Aria flow cytometry (BD Biosciences) into CD38- and CD38+ fractions. Leukemic Ph+ cells in the two separated populations were determined using the FISH technique with a dual-fusion dual-color *BCR-ABL1* probe (Vysis, Abbot, Downers Grove, IL, USA). [20,23] At least 1000 cells were counted from each cell fraction.

### Clinical data

At dg, patients were subjected to full clinical examination, including spleen size assessment by palpation. The CML risk status was evaluated according to the Sokal and Euro scoring systems.[7,13,14] Laboratory assessments both at dg and during follow-up included complete blood counts comprising white blood cell (WBC) counts and differential counts, red blood cell (RBC) counts, hemoglobin, hematocrit and red blood cell indices and platelet counts. In addition, BM morphology analysis and karyotyping were performed at dg and during follow-up.

### Statistical analysis

GraphPad Prism 6.0 and SPSS 22 were used for statistical analysis. Unpaired t-tests, chi-square tests, and one-way ANOVAs were applied when appropriate. P-values <0.05 were considered significant.

## Results

### Twenty-one percent of patients had no decline in *BCR-ABL1* transcript levels during the first month of TKI therapy

**Fig 1:**
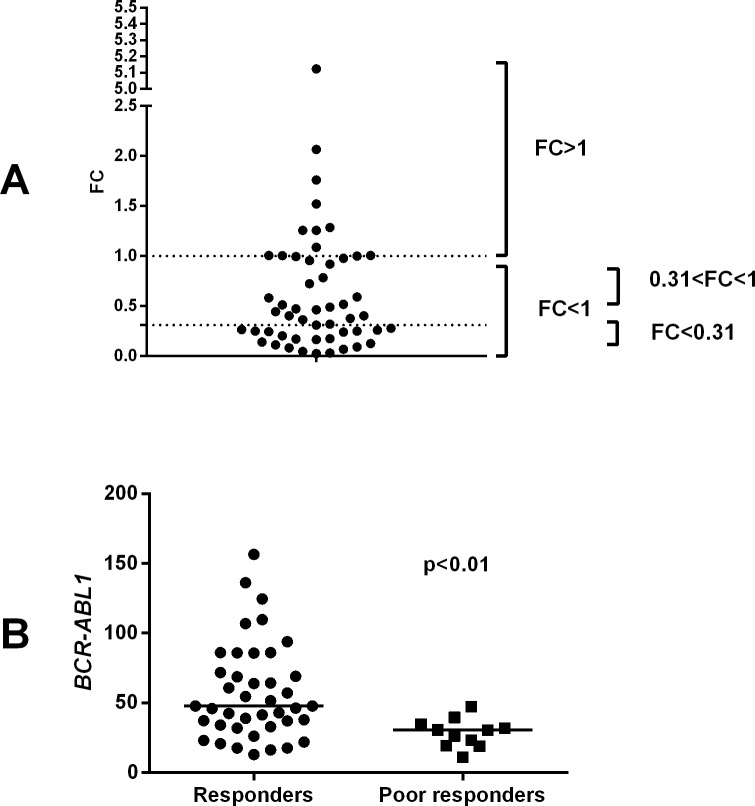
A proportion of CML patients do not respond during the first month of TKI therapy. A) The 1-month *BCR-ABL1* transcript value was divided by the *BCR-ABL1* transcript value at dg, and *poor responders* were identified as patients with fold change (FC) >1 (no decrease in the *BCR-ABL1* transcript level after 1 month). The patients with an FC lower than 1 were defined as *responders*. Furthermore, we used the median FC (0.31) of the responders to divide these patients into an *intermediate responders* group (0.31<FC<1) and *good responders* group (FC<0.31). B) The initial *BCR-ABL1* transcript level at dg was significantly lower for the *poor responders* than for the *responders* (p<0.01). Statistical significance was analyzed with an unpaired two-tailed t-test, and median values are noted with lines.

Patients were first divided into two groups based on the decline of *BCR-ABL1* transcripts at 1 month compared to diagnostic values. Patients with a fold change (FC) higher than 1 (i.e., no decrease in *BCR-ABL1* transcript values during the first month [FC>1]) were classified as *poor responders* (n = 11, 21% of total) and those lower than 1 (FC<1) as *responders* (n = 41). The median FC for the *responders* was 0.31, and this group of patients was further divided into *intermediate responders* (0.31<FC<1) and *good responders* (FC<0.31) (Fig 1A). In most of the *poor responders*, the *BCR-ABL1* transcript levels markedly increased during the first month of therapy when compared to the basal level at dg (Fig 1A).

### Poor responders had lower *BCR-ABL1* transcript levels at diagnosis (dg)

We next investigated factors possibly associated with increased disease burden at 1 month. Importantly, all patients were using the drug continuously, and no discontinuations or dose reductions were reported during the first month. First, we compared the initial *BCR-ABL1* transcript levels at dg between the *poor responder* and *responder* groups. Surprisingly, the *BCR-ABL1* transcript level in the *poor responder* group (median 30.5, range 10.9–47.2) was significantly lower than in the *responder* group (median 47.8, range 12.9–156.5) (p<0.01) (Fig 1B).

**Fig 2:**
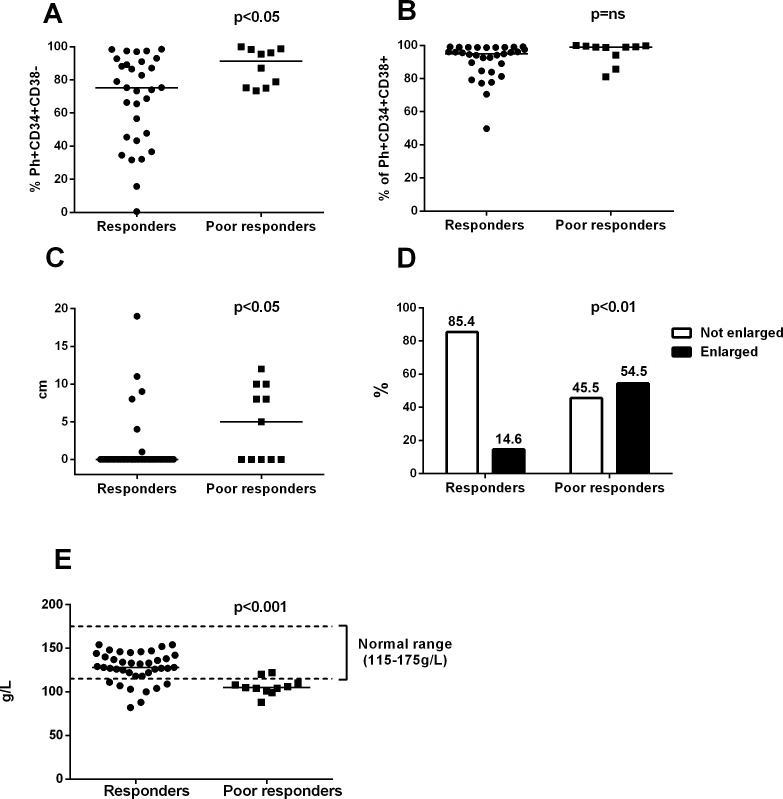
The *poor responders* have a higher percentage of Ph+CD34+CD38- cells and an enlarged spleen at diagnosis (dg). The proportion of Ph+ cells in the CD34+CD38- and CD34+CD38+ cell compartments was analyzed by sorting and FISH. **A)** The *poor responders* had a higher proportion of Ph+CD34+CD38- cells at dg when compared to the *responder* group (p<0.05). **B)** The proportion of Ph+CD34+CD38+ cells at dg. **C)** Difference in the size of the spleen (measured as the palpable part under the costal margin) between the *poor responder* and *responder* groups (p<0.05). **D)** Proportion of patients with enlarged spleen (pchi square <0.01). **E)** Hemoglobin levels in *poor responders* and *responders*. Statistical significance was analyzed with an unpaired two-tailed t-test, and median values are noted with lines. In panel D, the chi-square test was used.

As the use of different reference genes (*GUS* and *ABL*) may impact the *BCR-ABL1* transcript level at dg,[22] we divided the patients into two groups based on the reference gene used. We observed that the cases analyzed with the *ABL* gene had a significantly higher *BCR-ABL1* transcript level than the patients analyzed with *GUS* (*BCR-ABL1/ABL* median level 54.5% [range 17.5–156.6] vs. *BCR-ABL1/GUS* median level 34.8% [range 10.9–124.6] (p<0.05). As *GUS* was the reference gene in the majority of cases (n = 33), we also compared the diagnostic phase *BCR-ABL1* transcript levels between *poor responders* and *responders* in *GUS* control gene patients. Interestingly, we observed that the difference between the two groups remained significant, and *poor responders* had lower initial *BCR-ABL1* transcript values (median 30.5%, range 10.9–47.2) compared to *responders* (median 43.6%, range 12.9–124.6) (p<0.05) (S1 Fig). In the following paragraphs, we report the data from the whole patient cohort, including patients with either the *GUS* and *ABL* control genes. However, patients with the *GUS* control gene were also analyzed separately and showed similar results, and these are reported in the S1–S7 Figs. Supplemental figures showing only patients with the *GUS* control gene are enumerated identically to the corresponding main figures (i.e. S2A Fig corresponds Fig 2A). It should be noted that in each individual patient, only one control gene (either *GUS* or *ABL*) was used during the diagnostic and follow-up molecular analysis.

### Poor responders had a higher Ph+CD34+CD38- burden, an enlarged spleen, and a lower hemoglobin level at the time of dg

When we analyzed Ph positive (Ph+) putative leukemic stem cells (Ph+ CD34+CD38-) in the BM at dg, we found that the proportion out of all CD34+CD38- cells was significantly higher in *poor responders* (median 91.3%, range 73.4–100) than in *responders* (median 75.3%, range 0.6–98.5) (p<0.05) (Fig 2A and S2A Fig for the *GUS*-only result). The percentage of Ph+ CD34+CD38+ progenitor cells did not differ between the two groups (99.0 vs. 94.9%, p>0.05) (Fig 2B and S2B Fig).

Further analysis of clinical variables at dg revealed that the spleen was more frequently enlarged in the *poor responders* (6/11; 54.5%, median size 5 cm below costal margin) compared to the *responders* (6/41; 14.5%, median 0 cm below the costal margin) (t-test p<0.05; chi-square test p<0.01) (Fig 2C and 2D and S2C and S2D Fig). Moreover, hemoglobin levels were significantly lower in the *poor responders* (median 105g/L, range 88–122g/L) than in the *responders* (128g/L, range 82–154g/L) (p<0.001) (Fig 2E and S2E Fig).

Blasts, basophils, and total leukocyte and lymphocyte counts showed no difference between the two groups either at dg or during follow-up (3, 6, 12, and 18 months). Similarly, other factors, such as the type of TKI used, age, sex, and Euro or Hasford scores, were equal between the two groups *(*S1 and S2 Tables). All 11 *poor responders* were treated with hydroxyurea prior to TKIs, whereas only 17/41 (41.5%) of the *responders* had been treated with hydroxyurea (pchi square <0.01).

### Slower eradication of Ph+CD34+CD38- and Ph+CD34+CD38+ cells in the poor responders

**Fig 3:**
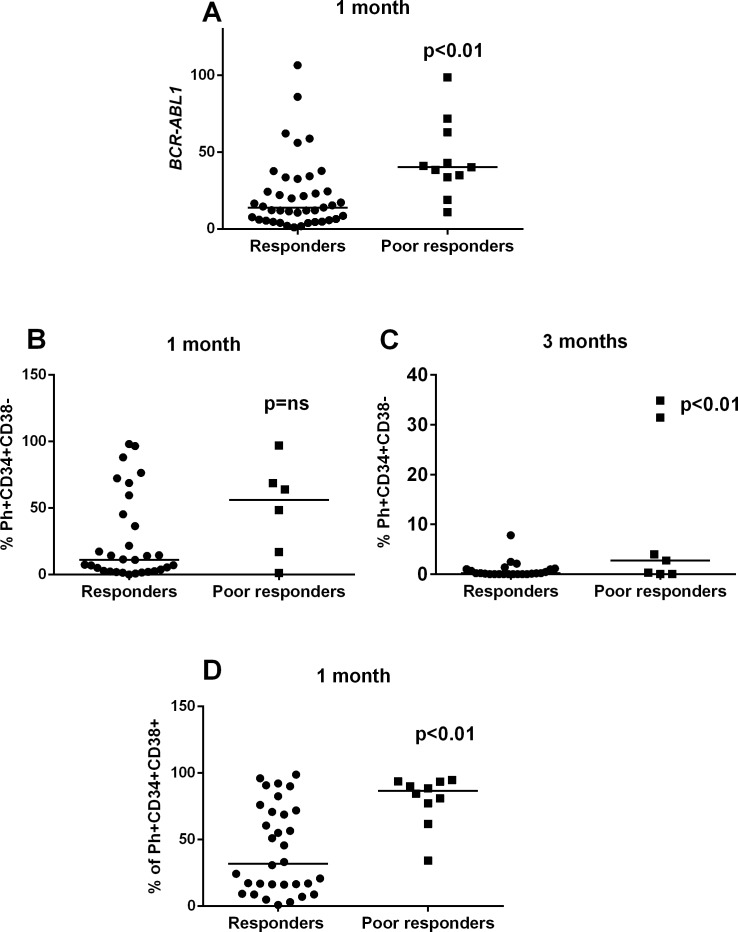
The Ph+CD34+CD38- cells are eradicated more slowly in *poor responders*. **A)** At 1 month, the *BCR-ABL1* transcript level was significantly higher in *poor responders* (median 40.2%, range 11–98.7) compared to *responders* (median 13.9%, range 0.9–106.6) (p<0.01) **B)** At 1 month, the proportion of Ph+CD34+CD38- cells in *poor responders* (median 56.2%, range 1.1–97) and *responders* (11.1%, range 0–98.0) **C)** At 3 months, *poor responders* had a significantly higher proportion of Ph+CD34+CD38- cells (median 2.8%, range 0–34.9) versus responders (0.2%, range 0–7.8) (p<0.01). **D)** *Poor responders* have a significantly higher percentage of Ph+CD34+CD38+ cells at 1 month (median 86.5%, range 34.2–94.8) compared to *responders* (median 32.0%, range 0.9–98.8) (p<0.01). Statistical significance was analyzed with an unpaired two-tailed t-test, and median values are noted with lines.

One month after the start of treatment, the *BCR-ABL1* transcript level was notably increased in *poor responders* (median at dg 30.5%, and 40.2% at 1m; p = 0.04), while the *responders* had a marked decrease in the *BCR-ABL1* transcript level (median at dg 47.8%, and 13.9% at 1m; p<0.0001). As a result, the *BCR-ABL1* transcript level at 1 month was significantly higher in *poor responders* (median 40.2%, range 11–98.7), when compared to the *responders* (median 13.9%, range 0.9–106.6, p<0.01) (Fig 3A and S3A Fig). The 3- and 6-month time points followed the same trend (median *BCR-ABL1* transcript levels for 3 months were 4.7% and 0.7% for *poor responders* and *responders*, respectively; while levels for 6 months were 1.2% and 0.15% for *poor responders* and *responders*, respectively).

**Fig 4:**
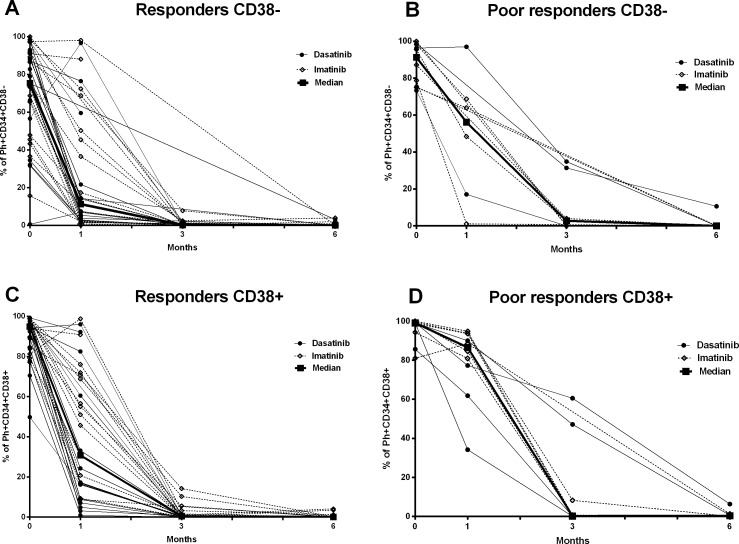
Ph+ in CD34+CD38- and CD34+CD38+ fractions decrease more slowly in the *poor responders*. The figure presents the proportions of Ph+ cells in CD34+CD38- (A and B) and CD34+CD38+ (C and D) fractions in *responders* (A and C) and *poor responders* (B and D) at the time of dg and during follow-up (1, 3, and 6 months). Imatinib-treated patients are presented with dashed lines and dasatinib-treated patients with solid lines. Medians in each group are marked with bold lines.

Consistent with the changes in *BCR-ABL1* transcript levels, the Ph+CD34+CD38- cell burden showed a dramatic decrease for *responders* after 1 month (median at dg 75.3%, and 11.1% at 1m; p<0.0001) (Fig 3B and S3B Fig) while a more modest decrease was observed in the *poor responders* (median at dg 91.3%, and 56.2% at 1m; p<0.05, Fig 3B). Accordingly, the percentage of Ph+ CD34+CD38- in *poor responders* was higher than in *responders* at 1 and 3 months (1m, median 56.2% [range 1.1–97] vs. 11.1% [range 0–98], p = 0.11; 3m, median 2.8% [range 0–34.86] vs. 0.2% [range 0–7.8] p<0.01) (Fig 3B and 3C, Fig 4A and 4B, S3B and S3C Fig, S4A and S4B Fig). No significant differences were observed at 6 months.

Similarly, the Ph+CD34+CD38+ cells decreased rapidly in the *responder* group (median at dg 94.7%, and at 1m 32.0%, p<0.0001) (Fig 4C). Notably, the *poor responders* still had a high Ph+CD34+CD38+ cell pool at 1 month (median at dg 99%, and at 1m 86.5%; p<0.05) (Fig 3D). Accordingly, the percentage of Ph+CD34+CD38+ cells was significantly higher at 1 month in the *poor responders* than in the *responders* (1m, median 86.5% [range 34.2–94.8] vs. 31.9% [0.9–98.6], p<0.01 (Figs 3D, 4C and 4D and S3D, S4C and S4D Figs). No significant difference was observed at 6 months.

### All responders had <10% BCR-ABL1 transcript levels at 3 months despite having high initial BCR-ABL1 transcript levels

**Fig 5:**
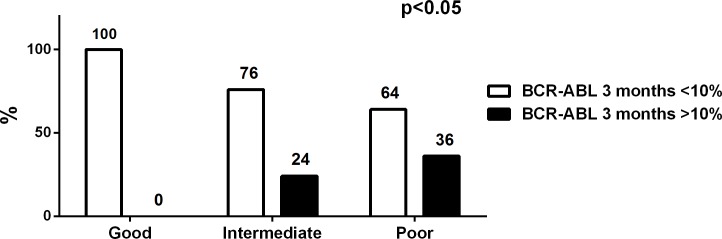
A third of the *poor responders* do not achieve a *BCR-ABL1* transcript level<10% at 3 months. Using the 3-month *BCR-ABL1* transcript 10% classification, 36% of the *poor responders* had *BCR-ABL1* transcripts>10%, whereas 0% of *good responders* (FC<0.31) and 24% of the *intermediate responders* (0.31<FC<1) were categorized into this group (see Fig 1A for classification of patients). Statistical significance was analyzed with a chi-square test.

We also compared our classification based on the FC to the 3-month response classification suggested in several studies (*BCR-ABL1* transcripts>10% as a sign of poor response).[7,15,16,19] We observed that all *good responders* (n = 17) had *BCR-ABL1* transcripts<10% at 3 months, whereas 24% (5/21 cases) of the *intermediate group* and 36% (4/11 cases) of the *poor responder* group had a *BCR-ABL1* transcript level higher than 10% at 3 months (p<0.01; Fig 5 and S5 Fig, patient subgroups are shown in Fig 1A and S1A Fig).

### Poor responders had lower MMR rates at 12 and 18 months compared to responders

**Fig 6:**
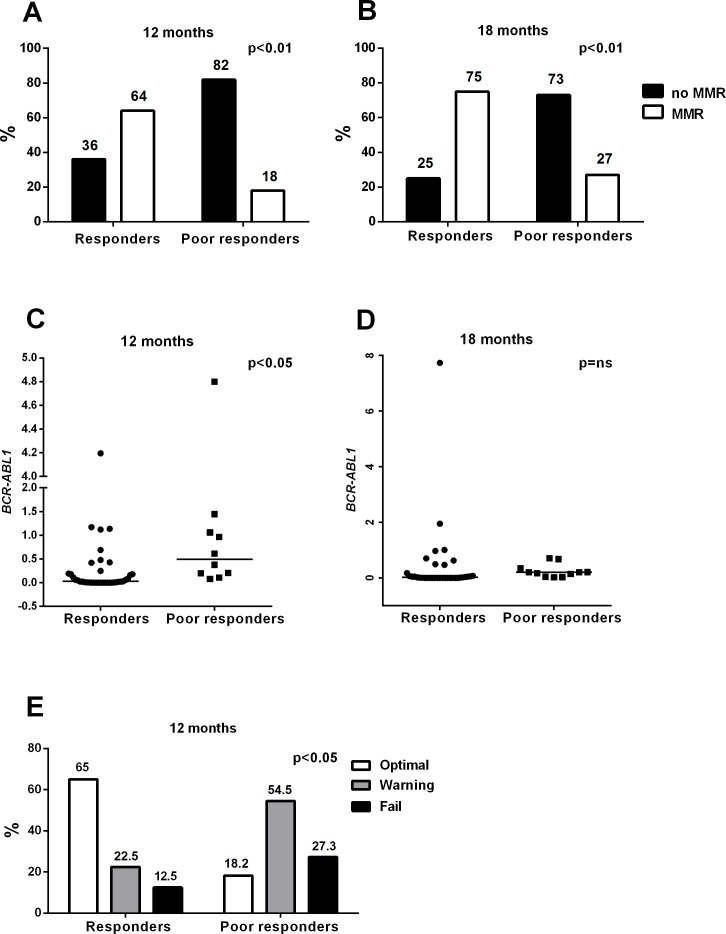
*Poor responders* have an inferior outcome at 12 and 18 months. **A-B)** The *poor responders* achieved MMR more seldom at 12 months (p<0.01) and 18 months (p<0.01) compared to *responders*. **C–D)** *Poor responders* had a higher *BCR-ABL1* transcript level at 12 months compared to the responders (p<0.05), whereas no difference was observed at 18 months. **E)** According to the ELN 12-month classification, the *poor responders* were significantly more often classified as warning and failures than the *responders* (p<0.05). In panels A, B, and E, statistical significance was analyzed with a chi-square test. In C and D, an unpaired two-tailed t-test was applied, and median values are noted with lines.

When later treatment responses were considered, we observed that the *poor responder* group had a significantly lower proportion of patients who achieved MMR at 12 months (only 2/11 [18%] of cases compared to 25/39 [64%] of cases in the *responders*; p<0.01, Fig 6A and S6A Fig). Furthermore, at 18 months, only 3/11 (27%) of the *poor responders* achieved MMR compared to 27/36 (75%) of the *responders* (p<0.01) (Fig 6B and S6B Fig). In addition, the *BCR-ABL1* transcript values at 12 months were higher for the *poor responders* when compared to the *responders* (0.5% vs. 0.03%; p<0.05) (Fig 6C and S6C Fig), and at 18 months, the value was 0.2% vs. 0.02% (p = ns) (Fig 6D and S6D Fig), respectively. Furthermore, according to the ELN 12-month classification[7] only 2 of the 11 *poor responders* (18%) achieved optimal response, while 6/11 cases were classified as warning (54%) and 3/11 cases as failures (27%). In contrast, in the *responders* (n = 41), 26 achieved optimal response (65%), 9 (22.5%) were categorized as warning, and 5 (12.5%) failed (p<0.0) (Fig 6E and S6E Fig).

**Fig 7:**
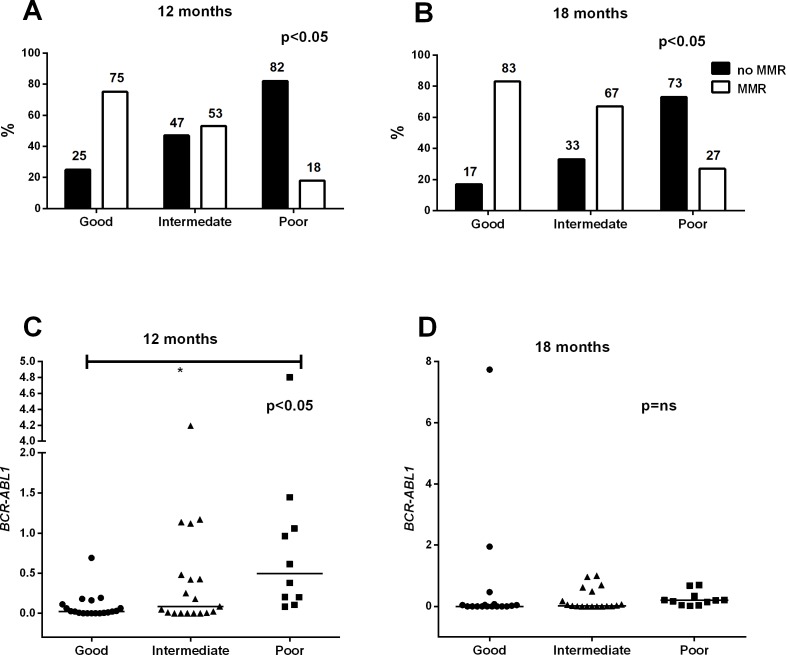
*Poor responders* have significantly worse long-term outcomes. The patients were divided into three groups based on the 1-month FC (*poor responders* FC>1, *intermediate* 0.31<FC<1, *good* FC<0.3; Fig 1A). **A–B)** The comparison of the three groups showed differences in the MMR rates at 12 (p<0.05) and 18 months (p<0.05). **C–D)** The *BCR-ABL1* transcript value at 12 and 18 months in the three response groups. In A and B, statistical significance was analyzed with a chi-square test. In C and D, an unpaired two-tailed t-test was applied, and median values are noted with lines.

When the *responders* were further divided into *good responders* (FC<0.31) and *intermediate responders* (0.31<FC<1) based on the median FC value of all responders (Fig 1A), we found that the *good responders* had the highest MMR rate at 12 months (15/20 cases; 75%) as well as at 18 months (15/18 cases; 83%). In addition, the *intermediate group* showed higher response rates (at 12 months, 10/19 cases [53%] reached MMR, and 12/18 cases [67%] achieved MMR at 18 months) than the *poor responders* (at 12 months, 2/11 cases [18%] reached MMR, and 3/11 cases [27%] achieved MMR at 18 months) (12m p<0.05; 18m p<0.05; Fig 7A and 7B and S7A and S7B Fig). The same trend was observed when comparing the *BCR-ABL1* transcript median values at 12 and 18 months (good responders, 0.02% and 0.0%, respectively; the intermediate responders, 0.09% vs. 0.02%, respectively; the poor responders, 0.5% and 0.2%, respectively) (12m p<0.05; 18m p = ns) (Fig 7C and 7D and S7C and S7D Fig).

### Poor responders had a significantly worse long-term treatment outcome

Long-term follow-up of the 11 *poor responders* showed that at 24 months, only 6/11 (54.5%) patients reached MMR (2 patients who had achieved MMR had switched from dasatinib to nilotinib) (Table 1). Furthermore, at 36 months, 7/10 patients (70%) had reached MMR (one had switched from dasatinib to nilotinib). The corresponding numbers for the *responders* were 27/31 (87.1%) at both 24 months and 36 months. A chi-square comparison between the responders and poor responders was significant at 24 months (p = 0.02), but at 36 months no differences were observed (Table 1).

**Table 1:** Differences in the MMR rates at 24 and 36 months between *responder* and *poor responder* groups.

	24 months molecular response			36 months molecular response		
	no MMR	MMR	Total	no MMR	MMR	Total
**Responders (percent)**	4 (12.9%)	27 (87.1%)	31 (100%)	4 (12.9%)	27 (87.1%)	31 (100%)
**Poor responders (percent)**	5 (45.5%)	6 (54.5%)	11(100%)	3 (30%)	7 (70%)	10 (100%)
**pchi-square**	**0.024**			**ns**		

A detailed characterization of the 11 *poor responders* revealed that 4 eventually stopped the treatment drug (imatinib n = 1, dasatinib n = 3), either due to toxicity (n = 3, 1 on imatinib and 2 on dasatinib) or treatment failure (on dasatinib) and switched to other TKIs (1 dasatinib patient switched to imatinib and 2 to nilotinib; the patient on imatinib discontinued the treatment).

## Discussion

Several trials have studied different possibilities to predict the response to TKI therapy at early time points in chronic phase CML, and most recommend 3 months as the earliest point [7,15–19]. As TKI therapy in general results in fast responses, we hypothesized that clinically meaningful response prediction would already be possible 1 month after the start of treatment. By comparing the *BCR-ABL1* transcript level at 1 month with the diagnostic value, we identified *poor-responding* patients who had no decline in *BCR-ABL1* transcript values. Importantly, regardless of the TKI used (either first- or second-generation drug) a significant proportion of these patients also failed to achieve optimal response at later time points.

In the current treatment algorithms, *BCR-ABL1* transcript level<10% at 3 months is the first treatment goal and a marker for early response.[7,15–19] However, this classification does not take into account patient-specific factors, such as initial *BCR-ABL1* transcript levels, which vary considerably between patients.[15,24] Therefore, the individual decline in the *BCR-ABL1* transcript level may be more informative than achieving the 10% landmark per se. Hanfstein and colleagues have studied the relative changes of *BCR-ABL1* transcript levels within the initial 3 months of therapy and defined good response as an achievement of a half-log reduction in *BCR-ABL1/GUS* values. Patients who failed to reach this half-log reduction at 3 months were at higher risk of disease progression.[15] Similarly, Branford et al. have shown that the early 3-month decline may be a critical prognostic factor when aiming to identify poor responders.[25] It should also be noted that our key findings remained similar when only patients in whom *GUS* has been used as a control gene were analyzed. Thus, the initial individual decline (or its absence) reflects disease biology, which is not dependent on the control gene used in the molecular analysis.

As the highest rate of progression to the accelerated and blast phases occurs during the first year of therapy, it is crucial to define poor-responding patients as early as possible to be able to modify their treatment in time. Therefore, response evaluation at the 1-month time point may have great significance for the subsequent outcome. In our patient cohort, we discovered that one-fifth (21%) of the patients failed to exhibit any decline in *BCR-ABL1* transcript levels during the first month of TKI therapy. Importantly, we confirmed that this was not due to poor compliance with the therapy, as all patients used full doses of assigned TKIs, according to their drug accountability logs in the clinical trial records.

All *responder* patients according to the 1-month FC classification in our study cohort achieved the 3-month 10% *BCR-ABL1* transcript level cut-off value [7,15,16,19], whereas 36% of the *poor responders* failed to achieve this despite their lower initial *BCR-ABL1* transcript value. In addition, according to the ELN recommendations, at the 12-month time point, over 80% of *poor responder* patients fell into the warning or failure categories, as they had not reached MMR.[7] Similarly, only 27% of these patients achieved MMR at 18 months. This was not related to the type of the TKI used, as both imatinib- and dasatinib-treated patients were represented in equal proportions. No nilotinib-treated patients were in the *poor responder* group, but only 5 such patients were evaluated. When MMR rates were evaluated at later time points (24 and 36 months), the *poor responders* still had inferior outcomes, although in 36% of the cases the treatment protocol had been changed. This clearly demonstrates that the *poor responders* are more likely to have a long-term poor response.

These results may suggest that the disease biology in the *poor responders* is different than in the well-responding patients. Higher initial *BCR-ABL1* transcript levels would have been a reasonable explanation for the slow response, but, to our surprise, the *poor responder* patients even had a lower median *BCR-ABL1* transcript level at dg when compared to the *responder* group. Age, total WBC, and blast or basophil counts did not differ between the groups. Interestingly, >50% of *poor responder* patients had splenomegaly at dg compared to the *responder* group, where palpable spleen was only discovered in <15% of the cases. Despite the many modern advances in diagnostic technology platforms, spleen size still seems to hold great importance when diagnosing and staging CML. It has been used in all past and current staging systems, such as in the Sokal, Euro, and EUTOS scores.[13,14,26]

In addition to an enlarged spleen, the *poor responders* had a higher proportion of Ph+CD34+CD38- cells in the hematopoietic stem cell compartment. The increased amount of Ph+ stem cells could partly explain the resistance observed in the *poor responders*, as it has been previously shown that quiescent CML stem cells are resistant to TKI therapy.[27–29] In addition, hemoglobin was observed to be lower in the *poor responders* at dg. These three factors taken together (spleen size, hemoglobin and Ph+ stem cells) may better represent the total tumor load in the body than the initial *BCR-ABL1* transcript level, and they therefore may explain the slower response to therapy. However, as we noticed that half of our *poor responders* had used the more potent second-generation TKI (dasatinib), it could also be that the enlarged spleen does not only imply a higher total tumor burden but also reflects issues with other biological aspects, such as aberrant signalling pathways that are activated and not well inhibited by the current TKIs. Interestingly, in the *poor responder* group, the disappearance of leukemic Ph+CD34+CD38+ cells was especially slow when compared to the *responder* group. As Ph+CD34+CD38+ cells are a rapidly dividing cell population, it could be that the initial increase in the *BCR-ABL1* transcript levels in the *poor responder* group mirrors the inefficiency of the TKI therapy used to inhibit this cell population. Of note, the *poor responders* had been treated significantly more often with hydroxyurea than the responding patients. Despite the hydroxyurea, these patients still responded poorly, most likely due to a higher tumor burden that was represented, for example, in the form of splenomegaly and increased leukemic stem cell burden. It has been previously observed that hydroxyurea can induce cytogenetic responses in some patients,[30,31] and therefore its use does not explain slower treatment responses in our patients.

In conclusion, our results suggest that the early *in vivo* response to TKI therapy after 1 month appears predictive for later treatment results. Patients who do not show any decline in the *BCR-ABL1* transcript levels during the first month of TKI therapy (i.e., *poor responders*) may have a different disease biology or significantly higher tumor burden. Thus, early response evaluation is warranted in prospective clinical trials.

## Supporting Information

S1 Fig Comparison of *GUS* and *ABL* control genes. A) The initial *BCR-ABL1* transcript value at dg. Patients with the *GUS* and *ABL* control genes are highlighted with different symbols. B) The patients in whom the *ABL* gene was used as the reference gene at dg had a significantly higher *BCR-ABL1* transcript value than the patients whose reference gene was *GUS* (p<0.05). C) If only cases in which *GUS* was used as a reference gene were considered, the initial *BCR-ABL1* transcript value was still lower in the *poor responder* group (p<0.05). Statistical significance was analyzed with an unpaired two-tailed t-test, and median values are noted with lines. D) Division of the patient groups based on the fold-change values (only patients with *GUS* control gene are presented). The 1-month *BCR-ABL1* transcript value was divided by the *BCR-ABL1* transcript value at dg, and *poor responders* were identified as patients with FC>1 (no decrease in *BCR-ABL1* transcript value after 1 month). The patients with an FC lower than 1 were defined as *responders*. Furthermore, we used the median FC (0.22) of the responders to divide these patients into an *intermediate response* group (0.22<FC<1) and *good response* group (FC<0.22).
(TIF)

S2 Fig The *poor responders* have enlarged spleen at dg (shown are patients with *GUS* as a control gene). A) The proportion of Ph+ cells in the CD34+CD38- and CD34+CD38+ cell compartments was analyzed by sorting and FISH. The *poor responders* tend to have a higher proportion of Ph+CD34+CD38- cells at dg when compared to the *responder* group (p = 0.08). B) No difference was observed in the proportion of Ph+CD34+CD38+ cells at dg. C) Difference in the size of the spleen (measured as the palpable part under the costal margin) between the *poor responder* and *responder* groups (p<0.001). D) Proportion of patients with enlarged spleen (pchi square = 0.001). E) Hemoglobin levels in *poor responders* and *responders* (p<0.001). Statistical significance was analyzed with an unpaired two-tailed t-test, and median values are noted with lines. In panel D, a chi-square test was used.
(TIF)

S3 Fig The Ph+CD34+CD38- cells are more slowly eradicated in *poor responders* (patients with *GUS* as a control gene). A) At 1 month, the *BCR-ABL1* transcript level was significantly higher in *poor responders* (median 40.2%, range 11–98.7) compared to *responders* (median 8.1%, range 0.99–58.7), (p<0.0001). B) At 1 month, the proportion of Ph+CD34+CD38- cells in *poor responders* (median 56.2%, range 1.1–97) and *responders* (14%, range 0–98.0). C) At 3 months, *poor responders* had a significantly higher proportion of Ph+CD34+CD38- cells (median 2.8%, range 0–34.9) versus responders (0.2%, range 0–2), (p<0.05). D) *Poor responders* have a significantly higher percentage of Ph+CD34+CD38+ cells at 1 month, median 86.5% (range 34.2–94.8) compared to *responders* (median 26.9%, range 3.1–98.8) (p<0.001). Statistical significance was analyzed with an unpaired two-tailed t-test, and median values are noted with lines.
(TIF)

S4 Fig Ph+ in CD34+CD38- and CD34+CD38+ fractions decrease more slowly in the *poor responders* (patients with *GUS* as a control gene). The figure presents the proportions of Ph+ cells in CD34+CD38- (A and B) and CD34+CD38+ (C and D) fractions in *responders* (A and C) and *poor responders* (B and D) at the time of dg and during follow-up (1, 3, and 6 months). Imatinib-treated patients are presented with yellow lines and dasatinib-treated patients with blue lines. Medians in each group are marked with green lines.
(TIF)

S5 Fig A third of the *poor responders* do not achieve *BCR-ABL1* transcript level<10% at 3 months (patients with *GUS* as a control gene). Using the 3-month *BCR-ABL1* transcript level 10% classification, 36% of the *poor responders* had *BCR-ABL1* transcript level>10%, whereas 0% of *good responders* (FC<0.22) and 24% of the *intermediate responders* (0.22<FC<1) were categorized into this group. Statistical significance was analyzed with a chi-square test.
(TIF)

S6 Fig *Poor responders* have inferior outcomes at 12 and 18 months (shown are patients with *GUS* as a control gene). A-B) The *poor responders* achieved MMR more seldom at 12 months (p<0.01) and 18 months (p<0.05) compared to *responders*. C-D) *Poor responders* had a higher *BCR-ABL1* transcript level at 12 months compared to the responders (p<0.05), whereas no difference was observed at 18 months. E) According to the ELN 12-month classification, the *poor responders* were significantly more often classified as warning and failures than the *responders* (p<0.05). In A, B, and E, statistical significance was analyzed with a chi-square test. In C and D, an unpaired two-tailed t-test was applied, and median values are noted with lines.
(TIF)

S7 Fig *Poor responders* have significantly worse long-term outcomes (shown are patients with *GUS* as a control gene). The patients were divided into three groups based on the 1-month FC (*poor responders* FC>1, *intermediate* 0.22<FC<1, *good* FC<0.22; S1D Fig). A–B) The comparison of the three groups showed differences in the MMR rates at 12 (p<0.05) and 18 months (p = 0.05) C–D) The *BCR-ABL1* transcript values at 12 and 18 months in the three response groups. In A and B, statistical significance was analyzed with a chi-square test. In C and D, an unpaired two-tailed t-test was applied, and median values are noted with lines.
(TIF)

S1 Table A. Characteristics of the *poor responder* patients based on the 1 month/dg *BCR-ABL1* transcript fold-change ratio. B. Characteristics of the *responders* based on the 1 month/dg *BCR-ABL1* transcript fold-change ratio (FC<1).
(DOCX)

S2 Table Differences between *responders* (FC<1) and *poor responders* (FC>1) in bone marrow (BM) and peripheral blood differential counts. (DOCX)
