# Supplementary figures and images for: Early BCR-ABL1 Transcript Decline after 1 Month of Tyrosine Kinase Inhibitor Therapy as an Indicator for Treatment Response in Chronic Myeloid Leukemia
Source: PLoS One. 2017 Jan 30;12(1):e0171041. doi: 10.1371/journal.pone.0171041 (PMC5279791; doi:10.1371/journal.pone.0171041)

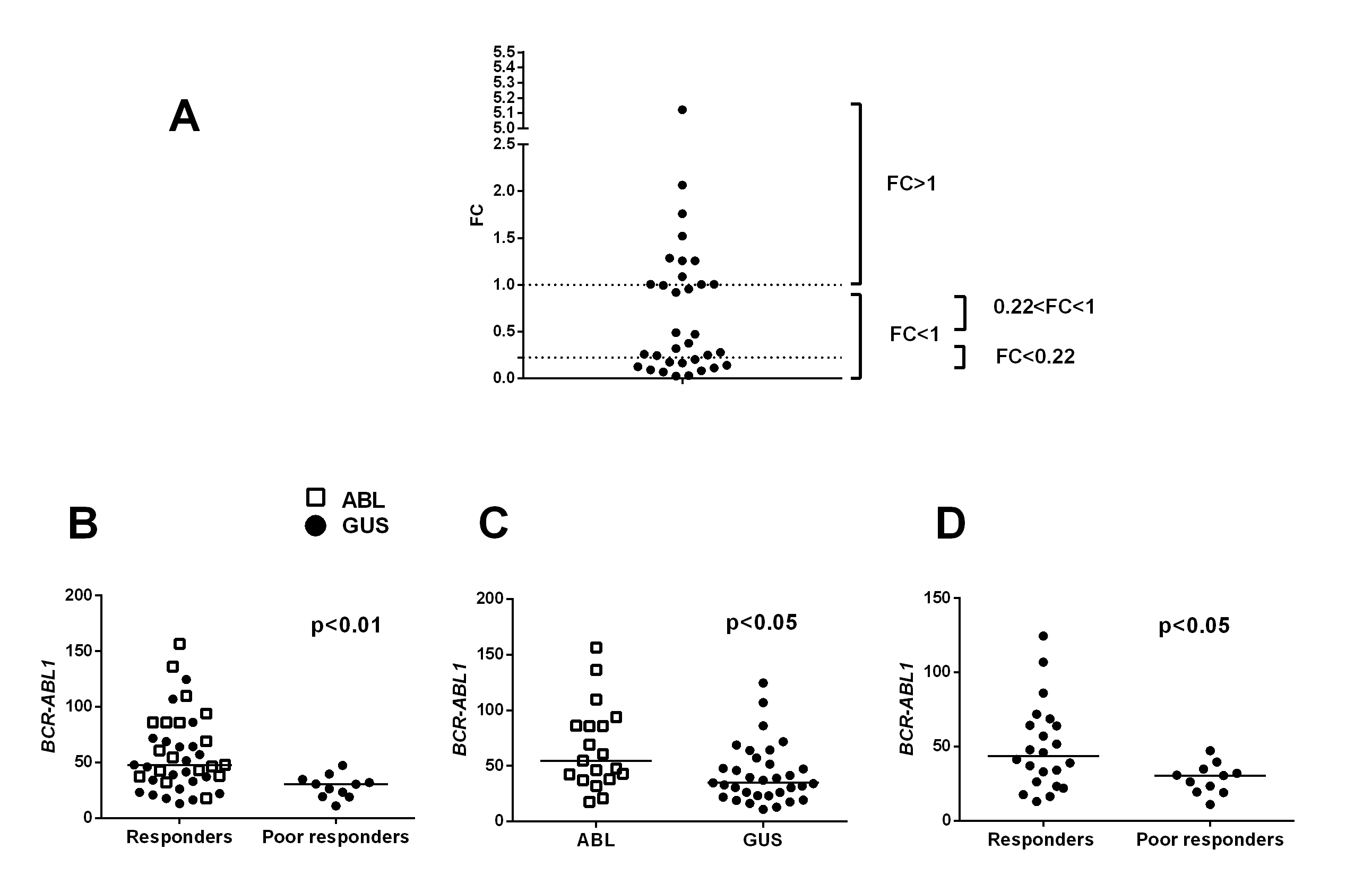

Supplement: S1 Fig — A) The initial BCR-ABL1 transcript value at dg. Patients with the GUS and ABL control genes are highlighted with different symbols. B) The patients in whom the ABL gene was used as the reference gene at dg had a significantly higher BCR-ABL1 transcript value than the patients whose reference gene was GUS (p<0.05). C) If only cases in which GUS was used as a reference gene were considered, the initial BCR-ABL1 transcript value was still lower in the poor responder group (p<0.05). Statistical significance was analyzed with an unpaired two-tailed t-test, and median values are noted with lines. D) Division of the patient groups based on the fold-change values (only patients with GUS control gene are presented). The 1-month BCR-ABL1 transcript value was divided by the BCR-ABL1 transcript value at dg, and poor responders were identified as patients with FC>1 (no decrease in BCR-ABL1 transcript value after 1 month). The patients with an FC lower than 1 were defined as responders. Furthermore, we used the median FC (0.22) of the responders to divide these patients into an intermediate response group (0.22<FC<1) and good response group (FC<0.22). (TIF) [file pone.0171041.s001.tif]

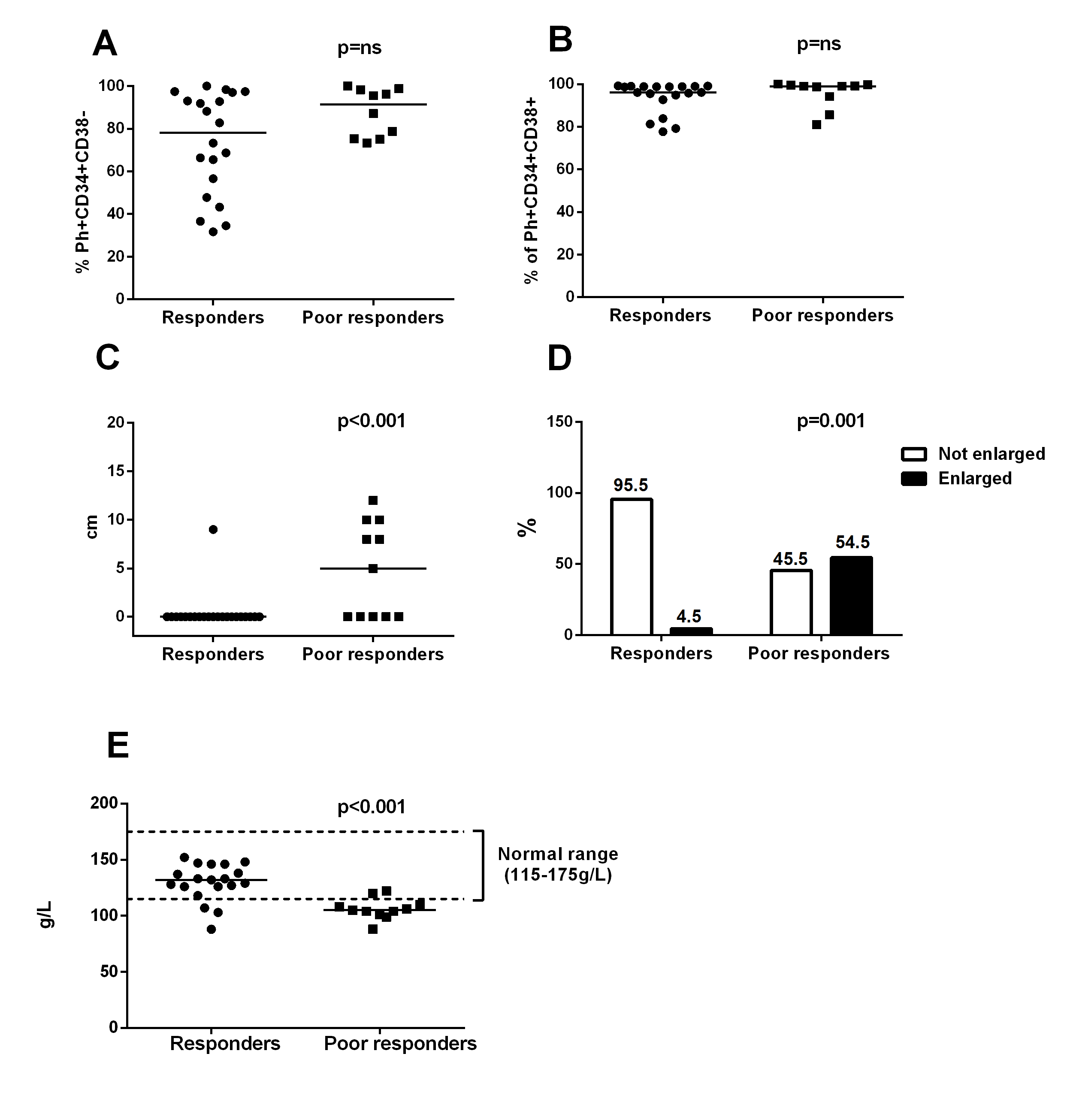

Supplement: S2 Fig — A) The proportion of Ph+ cells in the CD34+CD38- and CD34+CD38+ cell compartments was analyzed by sorting and FISH. The poor responders tend to have a higher proportion of Ph+CD34+CD38- cells at dg when compared to the responder group (p = 0.08). B) No difference was observed in the proportion of Ph+CD34+CD38+ cells at dg. C) Difference in the size of the spleen (measured as the palpable part under the costal margin) between the poor responder and responder groups (p<0.001). D) Proportion of patients with enlarged spleen (pchi square = 0.001). E) Hemoglobin levels in poor responders and responders (p<0.001). Statistical significance was analyzed with an unpaired two-tailed t-test, and median values are noted with lines. In panel D, a chi-square test was used. (TIF) [file pone.0171041.s002.tif]

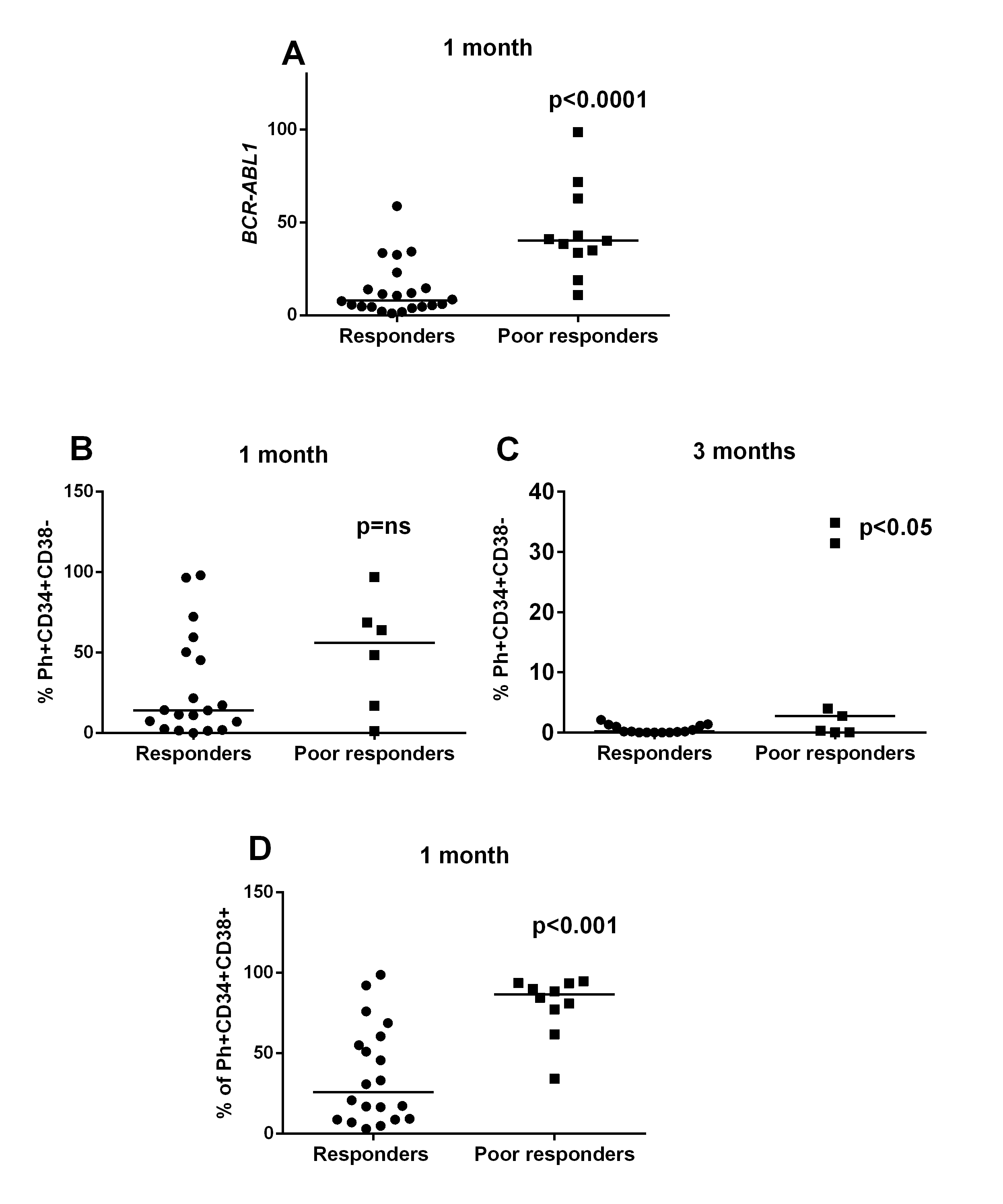

Supplement: S3 Fig — A) At 1 month, the BCR-ABL1 transcript level was significantly higher in poor responders (median 40.2%, range 11–98.7) compared to responders (median 8.1%, range 0.99–58.7), (p<0.0001). B) At 1 month, the proportion of Ph+CD34+CD38- cells in poor responders (median 56.2%, range 1.1–97) and responders (14%, range 0–98.0). C) At 3 months, poor responders had a significantly higher proportion of Ph+CD34+CD38- cells (median 2.8%, range 0–34.9) versus responders (0.2%, range 0–2), (p<0.05). D) Poor responders have a significantly higher percentage of Ph+CD34+CD38+ cells at 1 month, median 86.5% (range 34.2–94.8) compared to responders (median 26.9%, range 3.1–98.8) (p<0.001). Statistical significance was analyzed with an unpaired two-tailed t-test, and median values are noted with lines. (TIF) [file pone.0171041.s003.tif]

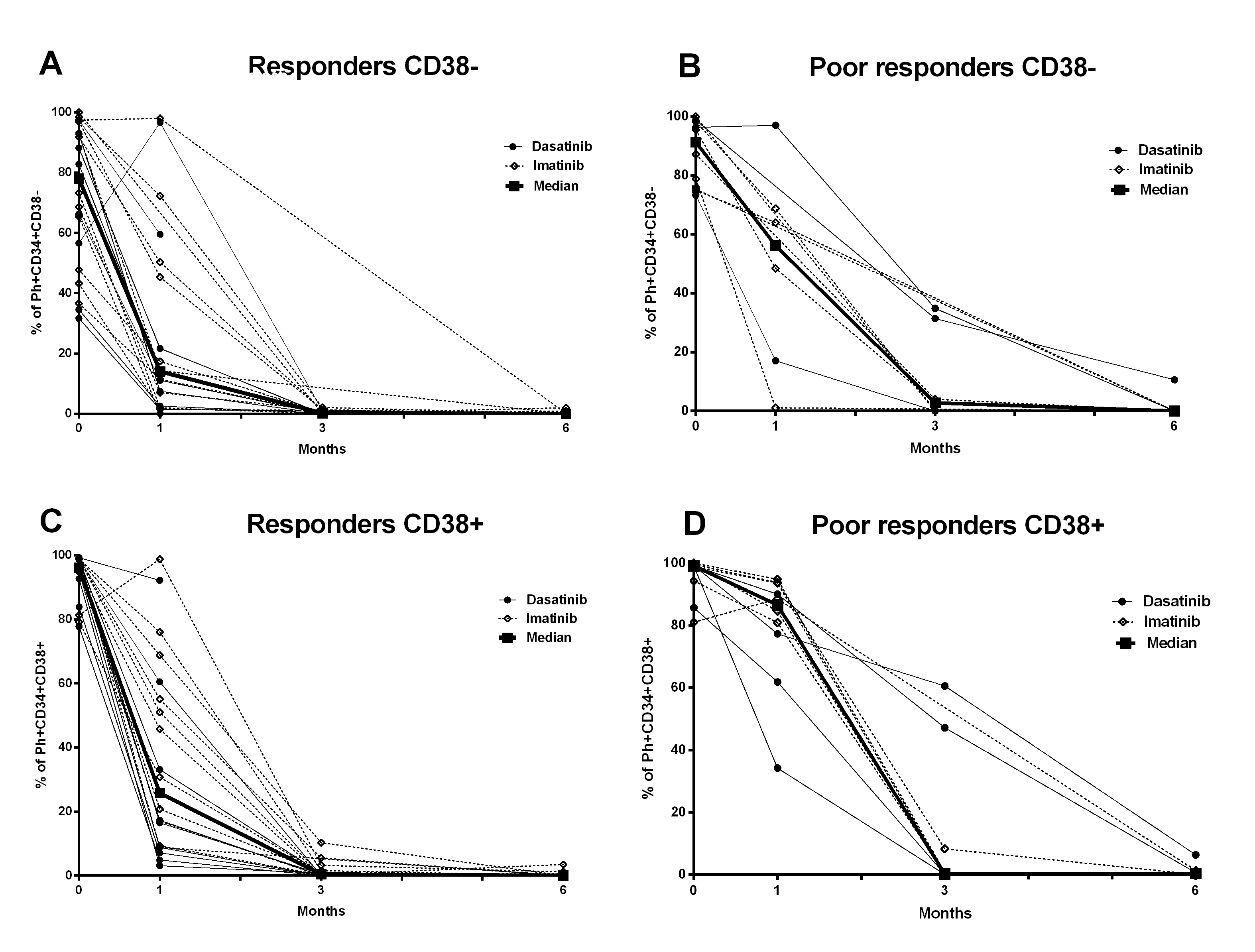

Supplement: S4 Fig — The figure presents the proportions of Ph+ cells in CD34+CD38- (A and B) and CD34+CD38+ (C and D) fractions in responders (A and C) and poor responders (B and D) at the time of dg and during follow-up (1, 3, and 6 months). Imatinib-treated patients are presented with yellow lines and dasatinib-treated patients with blue lines. Medians in each group are marked with green lines. (TIF) [file pone.0171041.s004.tif]

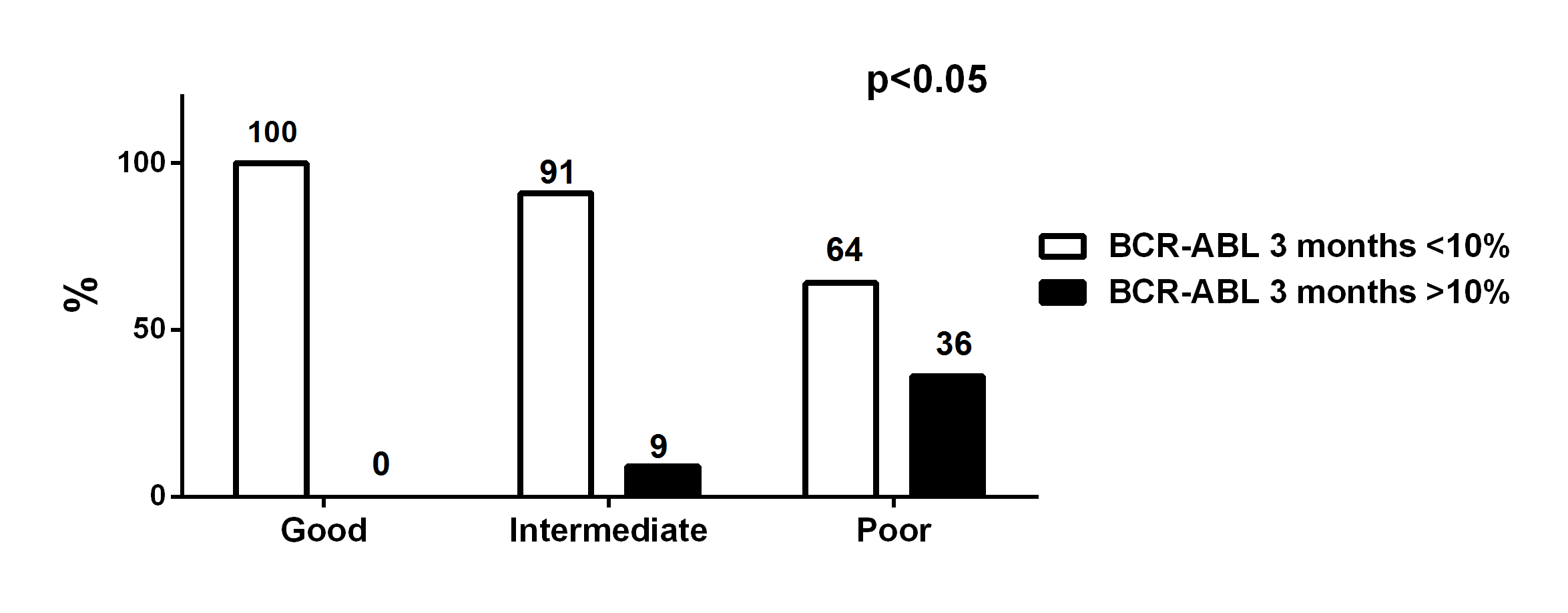

Supplement: S5 Fig — Using the 3-month BCR-ABL1 transcript level 10% classification, 36% of the poor responders had BCR-ABL1 transcript level>10%, whereas 0% of good responders (FC<0.22) and 24% of the intermediate responders (0.22<FC<1) were categorized into this group. Statistical significance was analyzed with a chi-square test. (TIF) [file pone.0171041.s005.tif]

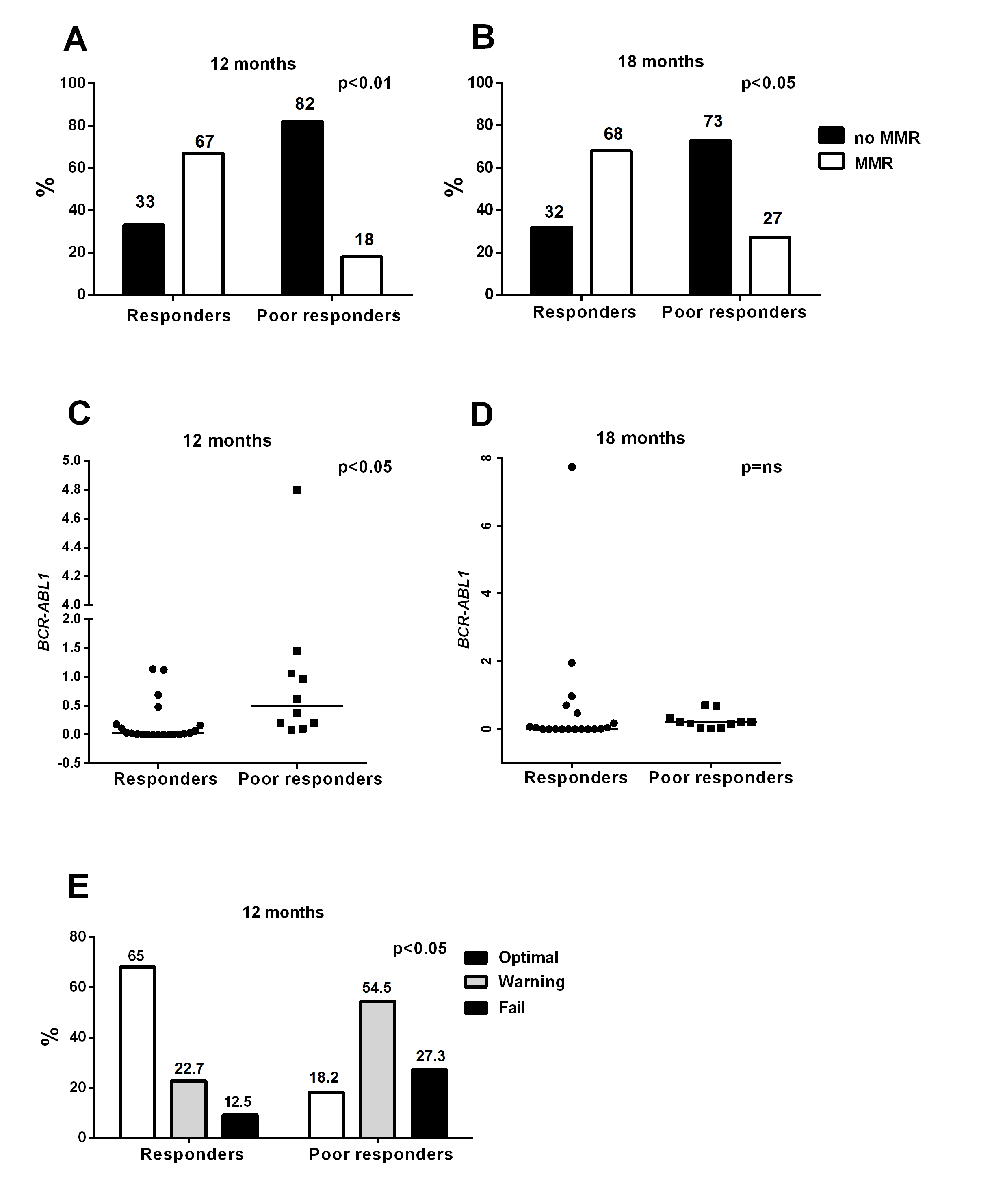

Supplement: S6 Fig — A-B) The poor responders achieved MMR more seldom at 12 months (p<0.01) and 18 months (p<0.05) compared to responders. C-D) Poor responders had a higher BCR-ABL1 transcript level at 12 months compared to the responders (p<0.05), whereas no difference was observed at 18 months. E) According to the ELN 12-month classification, the poor responders were significantly more often classified as warning and failures than the responders (p<0.05). In A, B, and E, statistical significance was analyzed with a chi-square test. In C and D, an unpaired two-tailed t-test was applied, and median values are noted with lines. (TIF) [file pone.0171041.s006.tif]

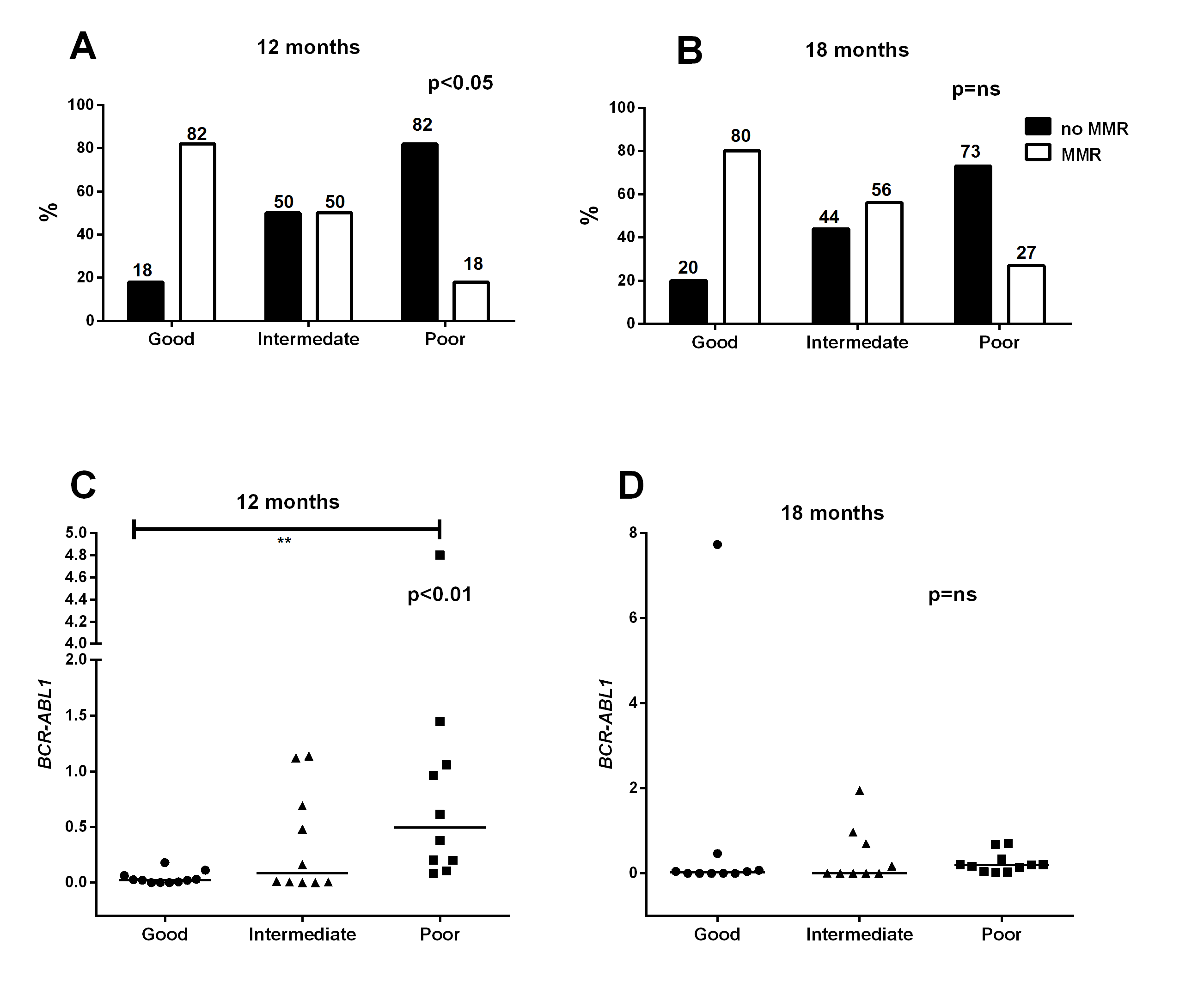

Supplement: S7 Fig — The patients were divided into three groups based on the 1-month FC (poor responders FC>1, intermediate 0.22<FC<1, good FC<0.22; S1D Fig). A–B) The comparison of the three groups showed differences in the MMR rates at 12 (p<0.05) and 18 months (p = 0.05) C–D) The BCR-ABL1 transcript values at 12 and 18 months in the three response groups. In A and B, statistical significance was analyzed with a chi-square test. In C and D, an unpaired two-tailed t-test was applied, and median values are noted with lines. (TIF) [file pone.0171041.s007.tif]
