# Supplementary material for: Early BCR-ABL1 Transcript Decline after 1 Month of Tyrosine Kinase Inhibitor Therapy as an Indicator for Treatment Response in Chronic Myeloid Leukemia
Source: PLoS One. 2017 Jan 30;12(1):e0171041. doi: 10.1371/journal.pone.0171041 (PMC5279791; doi:10.1371/journal.pone.0171041)
Supplement: S1 Table — A. Characteristics of the poor responder patients based on the 1 month/dg BCR-ABL1 transcript fold-change ratio. B. Characteristics of the responders based on the 1 month/dg BCR-ABL1 transcript fold-change ratio (FC<1). (DOCX) [file pone.0171041.s008.docx]

**S1A Table. Characteristics of the *poor responder* patients based on the 1 month/dg *BCR-ABL1* transcript fold change ratio.**

| **Pt** | **Drug** | **Sokal** | **Euro** | **Age at  diagnosis** | **Sex** | **Diagnosis  *BCR-ABL1*** | **1 month** |  | **FC  ratio** |
| --- | --- | --- | --- | --- | --- | --- | --- | --- | --- |
|  |  | **(risk group)** | **(risk group)** |  |  |  | ***BCR-ABL1*** | **Karyotyping** |  |
| 1 | IM | 0.57 | 796  (Intermediate) | 59 | Male | 19.3 | 98.7 | 46,XY,t,(9;22)(q34;q11)[20] | 5.1 |
|  |  | (Low) |  |  |  |  |  |  |  |
| 2 | IM | 0.68 | 428 | 43 | Male | 30.5 | 63 | 46,XY,t(9;22)(q34;q11)[27] | 2.1 |
|  |  | (Low) | (Low) |  |  |  |  |  |  |
| 3 | IM | 0.73 | 1053  (Intermediate) | 55 | Female | 23.2 | 41 | 46,XX T(9,22)(Q34,Q11) | 1.8 |
|  |  | (Low) |  |  |  |  |  |  |  |
| 4 | DA | 4.2 | 1839 | 61 | Male | 47.2 | 71.8 | 46,XY,t(9;22)(q34;q11)[20] | 1.5 |
|  |  | (High) | (High) |  |  |  |  |  |  |
| 5 | DA | 1.43 | 1048  (Intermediate) | 40 | Female | 26.2 | 33.7 | - | 1.3 |
|  |  | (High) |  |  |  |  |  |  |  |
| 6 | IM | 0.89  (Intermediate) | 866  (Intermediate) | 53 | Female | 32 | 40.2 | - | 1.3 |
| 7 | DA | 1.04  (Intermediate) | 1087  (Intermediate) | 64 | Male | 30.7 | 38.5 | 46,XY,t(9;22)(q34;q11)[18]/46,XY[2] | 1.3 |
| 8 | IM | 1.09  (Intermediate) | 1389  (Intermediate) | 62 | Female | 39.6 | 43 | 46,XX,t(9;22)(q34;q11)[5] | 1.1 |
| 9 | IM | 0.58 | 403 | 38 | Male | 34.8 | 35 | 46,XY,t(9:22)(q34:q11)[25] | 1 |
|  |  | (Low) | (Low) |  |  |  |  |  |  |
| 10 | DA | 0.85  (Intermediate) | 685 | 39 | Male | 10.9 | 11 | 46,XY,(q34;q11)[24]/46,XY[1] | 1 |
|  |  |  | (Low) |  |  |  |  |  |  |
| 11 | DA | 0.95 (Intermediate) | 745 | 29 | Female | 18.9 | 19 | 46,XX,t(9;22)(q34;q11)[15] | 1 |
|  |  |  | (Low) |  |  |  |  |  |  |

**S1B Table. Characteristics of the *responders* based on the 1 month/dg *BCR-ABL1* transcript fold change ratio (FC<1).**

| **Pt** | **Drug** | **Sokal**  **(risk group)** | **Euro**  **(risk group)** | **Age at diagnosis** | **Sex** | **Diagnosis *BCR-ABL1*** | **1 month *BCR-ABL1*** | **FC ratio** |
| --- | --- | --- | --- | --- | --- | --- | --- | --- |
| 12 | DAS | 1.14 | 1311 | 57 | Male | 85.9 | 85.9 | 0.9999 |
|  |  | (Intermediate risk) | (Intermediate risk) |  |  |  |  |  |
| 13 | IM | 0.65 | 182 | 43 | Male | 23.1 | 23 | 0.9938 |
|  |  | (Low risk) | (Low risk) |  |  |  |  |  |
| 14 | NIL | 0.66 | 870 | 50 | Female | 17.5 | 17.1 | 0.97 |
|  |  | (Low risk) | (Intermediate risk) |  |  |  |  |  |
| 15 | DAS | 0.75 | 287 | 45 | Female | 34.1 | 32.6 | 0.95 |
|  |  | (Low risk) | (Low risk) |  |  |  |  |  |
| 16 | IM | 4.21 | 2066 | 74 | Male | 63.9 | 58.7 | 0.92 |
|  |  | (High risk) | (High risk ) |  |  |  |  |  |
| 17 | IM | 0.8 | 1002 | 60 | Female | 136.2 | 106.6 | 0.78 |
|  |  | (Intermediate risk) | (Intermediate risk) |  |  |  |  |  |
| 18 | IM | 1.57 | 1810 | 64 | Male | 86 | 62.1 | 0.72 |
|  |  | (High risk) | (High risk) |  |  |  |  |  |
| 19 | DAS | 0.82 | 1242 | 51 | Female | 37.2 | 22 | 0.59 |
|  |  | (Intermediate risk) | (Intermediate risk) |  |  |  |  |  |
| 20 | NIL | 0.76 | 970 | 55 | Male | 20.7 | 12.1 | 0.58 |
|  |  | (Low risk) | (Intermediate risk) |  |  |  |  |  |
| 21 | DAS | 1.4 | 970 | 63 | Female | 32 | 16.6 | 0.52 |
|  |  | (High risk) | (Intermediate risk) |  |  |  |  |  |
| 22 | IM | 0.8 | 791 | 58 | Male | 109.8 | 56.1 | 0.51 |
|  |  | (Intermediate risk) | (Intermediate risk) |  |  |  |  |  |
| 23 | IM | 0.79 | 873 | 67 | Male | 68.7 | 33.6 | 0.49 |
|  |  | (Low risk) | (Intermediate risk) |  |  |  |  |  |
| 24 | DAS | 1.22 | 858 | 44 | Female | 16.3 | 7.7 | 0.47 |
|  |  | (High risk) | (Intermediate risk) |  |  |  |  |  |
| 25 | NIL | 0.68 | 100 | 49 | Male | 42.9 | 19.8 | 0.46 |
|  |  | (Low risk) | (Low risk) |  |  |  |  |  |
| 26 | DAS | 0.81 | 953 | 68 | Male | 54.5 | 24.2 | 0.44 |
|  |  | (Intermediate risk) | (Intermediate risk) |  |  |  |  |  |
| 27 | NIL | 0.69 | 386 | 49 | Female | 60.7 | 24.4 | 0.4 |
|  |  | (Low risk) | (Low risk) |  |  |  |  |  |
| 28 | DAS | 0.9 | 707.9 | 69 | Female | 93.9 | 37.8 | 0.4 |
|  |  | (Intermediate risk) | (Low risk) |  |  |  |  |  |
| 29 | DAS | 0.81 | 1012 | 59 | Male | 37 | 13.9 | 0.38 |
|  |  | (Intermediate risk) | (Intermediate risk) |  |  |  |  |  |
| 30 | IM | 0.89 | 994 | 73 | Female | 42.4 | 15.4 | 0.36 |
|  |  | (Intermediate risk) | (Intermediate risk) |  |  |  |  |  |
| 31 | DAS | 0.67 | 204 | 41 | Female | 107 | 34.3 | 0.32 |
|  |  | (Low risk) | (Low risk) |  |  |  |  |  |
| 32 | IM | 1.39 | 1567 | 60 | Male | 69 | 21.4 | 0.31 |
|  |  | (High risk) | (High risk) |  |  |  |  |  |
| 33 | IM | 0.76 | 1177 | 51 | Female | 41.3 | 11.5 | 0.28 |
|  |  | (Low risk) | (Intermediate risk) |  |  |  |  |  |
| 34 | IM | 0.93 | 1106 | 72 | Male | 46.2 | 12.2 | 0.26 |
|  |  | (Intermediate risk) | (Intermediate risk) |  |  |  |  |  |
| 35 | IM | 13.22 | 2049 | 78 | Female | 33 | 8.5 | 0.26 |
|  |  | (High risk) | (Low risk) |  |  |  |  |  |
| 36 | NIL | 0.9 | 362 | 48 | Male | 47.8 | 11.9 | 0.25 |
|  |  | (Intermediate risk) | (Low risk) |  |  |  |  |  |
| 37 | IM | 0.72 | 376 | 44 | Male | 47.8 | 11.9 | 0.25 |
|  |  | (Low risk) | (Low risk) |  |  |  |  |  |
| 38 | IM | 1.45 | 1252 | 67 | Female | 21.9 | 5.4 | 0.24 |
|  |  | (High risk) | (Intermediate risk) |  |  |  |  |  |
| 39 | IM | 0.84 | 928 | 64 | Male | 156.5 | 37.6 | 0.24 |
|  |  | (Intermediate risk) | (Intermediate risk) |  |  |  |  |  |
| 40 | DAS | 0.61 | 328 | 46 | Male | 71.8 | 14.6 | 0.2 |
|  |  | (Low risk) | (Low risk) |  |  |  |  |  |
| 41 | DAS | 0.64 | 452 | 47 | Female | 26.1 | 4.5 | 0.17 |
|  |  | (Low risk) | (Low risk) |  |  |  |  |  |
| 42 | DAS | 0.74 | 871 | 61 | Female | 38 | 6.5 | 0.17 |
|  |  | (Low risk) | (Intermediate risk) |  |  |  |  |  |
| 43 | IM | 0.69 | 791 | 57 | Female | 64.3 | 10.6 | 0.16 |
|  |  | (Low risk) | (Intermediate risk) |  |  |  |  |  |
| 44 | IM | 0.56 | 287 | 35 | Female | 12.9 | 1.8 | 0.14 |
|  |  | (Low risk ) | (Low risk) |  |  |  |  |  |
| 45 | IM | 0.58 | 496 | 40 | Male | 45.9 | 5.7 | 0.12 |
|  |  | (Low risk) | (Low risk) |  |  |  |  |  |
| 46 | DAS | 0.9 | 1242 | 61 | Female | 17.6 | 2 | 0.11 |
|  |  | (Intermediate risk) | (Intermediate risk) |  |  |  |  |  |
| 47 | IM | 0.6 | 369 | 44 | Male | 51.6 | 4.7 | 0.09 |
|  |  | (Low risk) | (Low risk) |  |  |  |  |  |
| 48 | IM | 0.89 | 707.9 | 74 | Male | 57.1 | 4.7 | 0.08 |
|  |  | (Intermediate risk) | (Low risk) |  |  |  |  |  |
| 49 | IM | 0.75 | 953 | 64 | Male | 86 | 6 | 0.07 |
|  |  | (Low risk) | (Intermediate risk) |  |  |  |  |  |
| 50 | DAS | 1.57 | 1313 | 56 | Female | 85.8 | 3.9 | 0.05 |
|  |  | (High risk) | (Intermediate risk) |  |  |  |  |  |
| 51 | DAS | 1.18 | 1118 | 71 | Female | 124.6 | 3.9 | 0.03 |
|  |  | (Intermediate risk) | (Intermediate risk) |  |  |  |  |  |
| 52 | DAS | 0.66 | 369 | 45 | Female | 38.9 | 1 | 0.03 |
|  |  | (Low risk) | (Low risk) |  |  |  |  |  |
