# Supplementary material for: Early BCR-ABL1 Transcript Decline after 1 Month of Tyrosine Kinase Inhibitor Therapy as an Indicator for Treatment Response in Chronic Myeloid Leukemia
Source: PLoS One. 2017 Jan 30;12(1):e0171041. doi: 10.1371/journal.pone.0171041 (PMC5279791; doi:10.1371/journal.pone.0171041)
Supplement: S2 Table — (DOCX) [file pone.0171041.s009.docx]

**S2 Table. Differences between *responders* (FC< 1) and *poor responders* (FC>1) in bone marrow and peripheral blood differential counts**

|  | **Dg**  **Resp.** | **Dg**  **Poor resp.** | **3m**  **Resp.** | **3m**  **Poor resp.** | **6m**  **Resp.** | **6m**  **Poor resp.** | **12m**  **Resp.** | **12m**  **Poor resp.** | **18m**  **Resp.** | **18m**  **Poor resp.** |
| --- | --- | --- | --- | --- | --- | --- | --- | --- | --- | --- |
| BM blasts (%)* | 2  (0-9) | 2  (1-4) | 1  (0-3) | 1.5  (1-5) | 1  (0-4) | 1  (0-1) | 1  (0-3) | 1  (0-2) | 2  (0-4) | 1.5  (0-4) |
| BM lymphocytes (%)* | 3.5  (1-10) | 2  (0- 13) | 27  (7-40) | 19  (14-38) | 26  (10-37) | 20.5  (6-29) | 16.5  (4-40) | 16  (1-39) | 18  (3-34) | 13.5  (1-42) |
| BM basophils (%)* | 2  (0-10) | 2.5  (0-5) | 0  (0-2) | 1  (0-1) | 0  (0-2) | 0  (0-1) | 0  (0-1) | 0  (0-0) | 0  (0-2) | 0  (0-2) |
| PB WBC (10^9^/L)* | 44.7  (2.4- 288.4) | 46.6  (5.6- 169.5) | 5  (2-8.8) | 4.6  (2.2- 7.6) | 5.6  (2.7- 12.3) | 4.6  (2.1-6.9) | 5.4  (2.7-10.8) | 5.95  (2.4-11.6) | 5.5  (3.2-11.7) | 5.35  (3-9.4) |
| PB relative lymphocyte count (%)* | 9  (2-35) | 6  (1-16) | 36  (3-70) | 30  (14-48) | 34  (17-73) | 33.5  (6-66) | 31  (0-62) | 27  (12-45) | 33  (20-67) | 34  (13-56) |
| PB absolute lymphocyte count (10^9^/L) * | 3.4  (0-11.7) | 2.7  (0.5-15.5) | 1.7  (0.1-4.4) | 1.0  (0.6-2.4) | 1.8  (0.7-5.6) | 1.6  (0.3-2.9) | 1.7  (0-5.1) | 1.3  (0.7-3.0) | 1.7  (0.8-5.2) | 1.5  (1.1-3.1) |
| *Median (minimum-maximum)  **Abbreviations:** Dg, diagnosis; m, months; BM, bone marrow; PB, peripheral blood; WBC, white blood cell | | | | | | | | | | |
